# Proteome profiling indicates a link between mitochondrial pathways and the host-microbial sensor ELMO1 following *Salmonella* infection

**DOI:** 10.1080/19490976.2025.2580708

**Published:** 2025-11-17

**Authors:** Sajan C Achi, Dominic McGrosso, Stefania Tocci, Isaac Amao, Baibaswata Saha, Stella-Rita Ibeawuchi, Ibrahim M. Sayed, David J Gonzalez, Soumita Das

**Affiliations:** a Department of Biomedical and Nutritional Sciences, University of Massachusetts - Lowell, Lowell, MA, USA; b Department of Pathology,University of California, San Diego, CA, USA; c Department of Pharmacology, University of California, San Diego, CA, USA; d Skaggs School of Pharmacy and Pharmaceutical Sciences, University of California, San Diego, CA, USA

**Keywords:** Microbial sensor, ELMO1, bacterial effector, SifA, macrophages, proteomics, mitochondrial dynamics, DRP1, mitochondrial fission

## Abstract

The host EnguLfment and cell MOtility protein 1 (ELMO1) is a cytosolic microbial sensor that binds bacterial effector proteins, including pathogenic effectors from *Salmonella* (*Salmonella* enterica serovar Typhimurium) and controls host innate immune signaling. To understand the ELMO1-regulated host pathways, we have performed liquid chromatography Multinotch MS3-Tandem Mass Tag (TMT) multiplexed proteomics to determine the global quantification of proteins regulated by ELMO1 in macrophages during *Salmonella* infection. Comparative proteome analysis of control and ELMO1-depleted murine J774 macrophages after *Salmonella* infection quantified more than 7000 proteins with a notable enrichment in mitochondrial-related proteins. Gene ontology enrichment analysis revealed 19 upregulated and 11 downregulated proteins exclusive to ELMO1-depleted cells during infection, belonging to mitochondrial functions, metabolism, vesicle transport, and the immune system. Seahorse analysis showed that *Salmonella* infection alters mitochondrial metabolism from oxidative phosphorylation to glycolysis-a shift significantly influenced by the depletion of ELMO1. Furthermore, ELMO1 depletion decreased the ATP rate index following *Salmonella* infection, indicating its importance in counteracting the effects of *Salmonella* on immunometabolism. Among the proteins involved in mitochondrial pathways, the mitochondrial fission protein DRP1 was significantly upregulated in ELMO1-depleted cells and ELMO1-KO mice intestine following *Salmonella* infection. Pharmacological inhibition of DRP1 identified the role of ELMO1-DRP1 pathway in the regulation of pro-inflammatory cytokine TNF-α following infection. The role of ELMO1 has been further characterized by a Proteome profiling of ELMO1-depleted macrophage infected with SifA mutant displayed the involvement of ELMO1-SifA in mitochondrial function, metabolism and host immune/defense responses. Collectively, these findings reveal a novel role for ELMO1 in modulating mitochondrial functions, potentially pivotal in modulating inflammatory responses.

## Graphical Abstract

**Figure:**
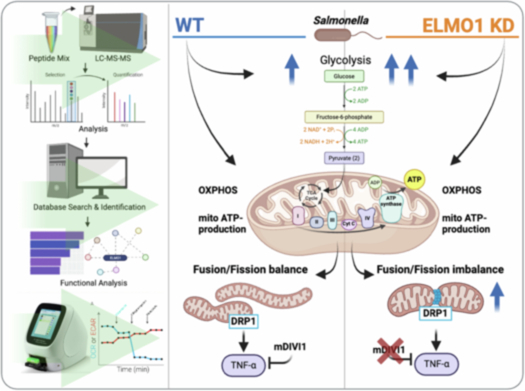

## Introduction

Host cellular responses coordinate during infections to maintain immune homeostasis. Infection with enteric pathogens generates inflammation and tissue damage, which leads to 4−6 million human deaths worldwide per year. Microbial sensing controls inflammation after recognizing invading pathogens and regulates the expression of proteins critical for disease outcome. Understanding the molecular mechanisms governing host‒pathogen interactions is vital for preventing and treating infectious diseases worldwide. To this end, mass spectrometry (MS) has become a powerful and effective approach in proteomics to better understand complex and dynamic host‒pathogen interactions in unbiased fashions. Recent breakthroughs in the proteomics field have included the use of isobaric labels termed tandem mass tags (TMTs). The TMT approach has been shown to yield data of high quantitative accuracy, particularly when LC‒MS3 level quantification using the synchronous precursor selection (SPS) is performed. *Salmonella* has been used as a model enteric bacterial pathogen, and the proteome profile of the bacteria has been extensively studied.^1-4^ In contrast, how the host proteome fluctuates during infection is less understood given the complexity of the host proteome and the greater range of proteins present. Previous studies using LC-MS/MS of RAW 264.7 macrophages after *Salmonella* infection identified the involvement of proteins in the production of antibacterial nitric oxide, the production of prostaglandin H2, and the regulation of intracellular trafficking.^5^ A global host phosphoproteome analysis after bacterial infection identified the kinase SIK2 as a central component of the host defense machinery upon *Salmonella* infection.^6^

Though host transcriptome profiling has been widely practiced in host‒pathogen interactions,^7^ only a few studies reported multiplex proteomic profiling of the host proteome upon infection. Proteomics has been used to study host protein profiles during infection,either studying the whole cell proteome^5^^,^^8^ or subsets of the host proteome such as Golgi networks^9^^,^^10^ and *Salmonella*-containing vacuoles (SCVs).^11^ To understand the host cellular pathways modified in macrophages after *Salmonella* infection, here, we have used tandem mass tags, where chemical labeling with combined samples reduces errors associated with run-to-run variations caused by temperature or column conditions.^12^

For microbial sensing, Pattern Recognition Receptors (PRRs) recognize pathogen-associated molecular patterns (PAMPs) or host-derived damage-associated molecular patterns (DAMPs) and activate inflammatory signals.^13-15^ Our published work identified Brain Angiogenesis Inhibitor1 (BAI1) as a new PRR which was initially identified as a receptor for apoptotic cells.^16^ The intracellular domain of BAI1 interacts with ELMO1 (Engulfment and cell motility protein 1), which associates with Dock180 (Dedicator of cytokinesis 180) to act as a bipartite guanine nucleotide exchange factor (GEF) for the small GTPase Rac1^16^ and the activated Rac1 facilitates the engulfment of bacteria. We have shown that ELMO1 is more than just a director of phagocytosis^17^ and involved in the internalization of enteric bacteria and generation of intestinal inflammation^18^; ELMO1 is elevated in patients with inflammatory bowel disease and induces inflammatory cytokines^19^^,^^20^ ELMO1 regulates autophagy induction and bacterial clearance during infection with *Salmonella*^21^; ELMO1 interacts with microbial sensor NOD2 and together with mutant NOD2 associated with Crohn's disease (CD) are unable to clear CD-associated bacteria.^22^ Interestingly, ELMO1 differentially regulates the immune response after sensing pathogens and interacts with the bacterial effectors containing a signature motif called WxxxE, which is absent in the commensals.^23^^,^^24^ The same work identified the *Salmonella* WxxxE-containing effector SifA interacts with ELMO1, and controls innate immune responses.

Here, we have used TMT-guided LC-MS3 proteomics to determine the global quantification of proteins regulated by ELMO1 in macrophages during *Salmonella* infection. Comparative proteome analysis of control and ELMO1-depleted murine macrophages (J774) after *Salmonella* (*SL*) infection revealed more than 7000 proteins and identified the mitochondrial pathway as the top candidate regulated by ELMO1. We also identified the differential proteome profile after infection of ELMO1-depleted macrophages with WT *SL* and *SifA* -mutant *Salmonella*. Together, we report an extensive comparative proteome profile of ELMO1-regulated host proteome components following *Salmonella* infection.

## Materials and methods

### Bacterial strains and growth conditions

*Salmonella enterica* serovar Typhimurium (strain *SL1344, abbreviated as SL*) and *sifA* mutant strains were used in this study following the culture methods described previously.^18^^,^^24^^,^^25^

### Cell culture and infection

Culturing and maintenance of control (C1) and ELMO1-depleted (E1) J774 macrophages (J774) were described previously^18^^,^^24^. For infection, approximately 8 × 10^6^ cells were plated onto a 100 mm dish, and the cells were infected with SL at a multiplicity of infection (moi) of 1:10 for 1 h. Extracellular bacteria were killed by gentamicin treatment (500 µg/mL) for 90 min at 37 °C, followed by treatment with low-dose gentamicin (50 µg/mL) for 3.5  h. The cell pellets were collected and stored at -80 °C until processing.

### Preparation of samples for multiplex proteomics

A Q500 sonicator (Qsonica) equipped with a 1.6 mm microtip at 20% amplitude was used to lyse the samples in lysis buffer (200 µL) as described previously.^26^ The lysis buffer was prepared with 7% SDS, 6 M urea, 50 mM TEAB, pH 8.1, and complete protease inhibitor and PhosStop from Roche. A total of 500 mM DTT at 47 °C for 30 min and 500 mM iodoacetamide (IAA) in the dark at room temperature (RT) for 45 min were used for reduction and alkylation of the samples as previously described.^26^ The quenching was performed with 500 mM DTT at RT for 5 min. The samples were acidified with 12% phosphoric acid and mixed in a 7:1 ratio with binding buffer composed of 90% methanol and 50 mM TEAB, pH 7.1. Finally, the samples were loaded into S-Trap columns (Protifi). A total of 5  µg of sequence-grade trypsin (Promega) in 50 mM TEAB for 3 h at 47 C was used for on-column digestion. For sequential elution of the peptides, we used 50 mM TEAB, 50% acetonitrile and 5% formic acid, followed by drying in a speed-vac. In the next step, C18 Sep-Paks (Waters) were used for desalting and elution with 40% and 80% acetonitrile solutions containing 0.5% acetic acid. The peptide concentration was determined using the Pierce protein estimation kit. To control for run-to-run variation, 10 µg of the sample was pooled for “bridge” channels as previously described.^26^^,^^27^ Each sample was resuspended in 30% dry acetonitrile in HEPES (200 mM, pH 8.5) for tandem mass tag labeling with TMT reagent (7 μl), as previously described.^28^ Labeling was performed at RT for 1 h and quenched with 8 μl of 5% hydroxylamine (Sigma). A total of 50 μl of 1% TFA was added to the labeled samples for acidification. After TMT labeling, each 10-plex experiment was combined, desalted using C18 Sep-Paks (Waters), and dried. The TMT manufacturer's channel contamination was regulated by reporting contamination values as mentioned by the VL312003-lot number. The labeled samples were subsequently subjected to basic pH reverse-phase liquid chromatography from Thermo Scientific (Ultimate 3000, with a Biobasic c18 column), and the fractions were collected as previously described,^26^^,^^29^ followed by drying. Finally, the samples were resuspended and placed in glass vials with 20 µL of 5% formic acid and 5% acetonitrile.

### LC-MS^3^ strategies

LC‒MS/MS/MS with an Orbitrap Fusion mass spectrometer from Thermo Scientific with an in-line EASY-nLC 1000 instrument was used to acquire the sample data as previously described.^26^^,^^29^ Briefly, the fractions were run on three-hour gradients starting at 3% acetonitrile and 0.125% formic acid and ending at 100% acetonitrile and 0.125% formic acid. To separate the peptides, we used an in-house packed column (30 cm × 100 μm inner diameter and 360 μm outer diameter) composed of 0.5 cm C4 resin (diameter = 5 μm), 0.5 cm C18 resin (diameter = 3 μm), and 29 cm C18 resin (diameter = 1.8 μm).^26^ For the MS1 spectrum acquisition, we performed in data-dependent mode with an Orbitrap survey (scan range of 500–1200 m/z) and a resolution of 60,000. 2 × 10^5^ automatic gain control (AGC) with a maximum ion injection time of 100 ms Top *N* was used with *N* = 10 for both MS2 and MS3 fragment ion analysis. The decision tree option was used for MS2 data collection. Ions carrying 2 charges were analyzed between 600 and 1200 m/z, and ions carrying 3 or 4 charges were analyzed between 500 and 1200 m/z as previously described, with an ion intensity threshold of 5 × 10^4^.^26^ The quadrupole at 0.5 Th was used for the isolation of selected ions, and collision-induced dissociation (CID) was used for fragmentation. Fragment ion detection and data centroiding occurred using the linear ion trap, where the rapid scan rate of the AGC target was 1 × 10^4^. TMT-based quantitation via MS3 fragmentation was performed using synchronous precursor selection. Up to 10 MS2 precursors were concurrently fragmented using High Energy Collisional Dissociation (HCD) fragmentation. Reporter ions were detected in the Orbitrap at a resolution of 60,000 and with a lower threshold of 110 m/z. The AGC was set to 1 × 10^5 with a maximum ion injection time of 100 ms. The data collected were centroided, and precursor ions outside of 40 m/z below and 15 m/z above the MS2 m/z were removed.

### Proteomic data analysis

To search the mass spectrometry data, we used Proteome Discoverer 2.5 software from Thermo Fisher Scientific against the reference proteome for *Mus musculus* downloaded from UniProt.com on 10 January 2021. The SEQUEST HT search algorithm was employed to align MS2 spectral data against theoretical peptides as previously described.^26^^,^^30^ The precursor tolerance was set to 50 ppm, and the fragment tolerance was set to 0.6 Da. For TMT labels on *N*-termini and lysine residues and for carbamidomethylation of cysteines, we specified the static modifications, and the dynamic modifications were set for the oxidation of methionine. A 1% false discovery rate cutoff was specified for the decoy database search.^31^ After filtering for quality (S/N > 10, Isolation Interference <25), we summed the remaining peptide spectral match abundances to the protein level. The average value for each protein divided by the median of all average protein values was used for normalization as previously described.^26^ A second normalization step was calculated where the abundance value for every protein per sample was divided by the median value for each channel that had been divided by the overall dataset median. To identify the differentially abundant proteins, the *π* score was used. A significance metric that incorporates both fold changes and traditional *p* value-based significance scores was determined through a Student's t test with or without Welch's correction, depending on the comparison.^26^^,^^32^

### Venn, enrichment, and network analysis

Venn diagrams were plotted using http://bioinformatics.psb.ugent.be/webtools/Venn/.

Enrichment analysis for biological processes, cellular components and molecular functions was performed using Database for Annotation, Visualization and Integrated Discovery (DAVID) v 2021^33^^,^^34^ and Enrichr.^35-37^ Graphs were constructed using Microsoft Office 365 and GraphPad Prism v 9.0.

### Seahorse XFp real-time ATP rate assay

Seahorse analysis of ATP production was performed using the Seahorse XFp Real-Time ATP Rate assay, following the manufacturer’s instructions. Briefly, C1 and E1 cells were seeded in Seahorse XFp Miniplate (Agilent Technologies, Santa Clara, CA, USA) at an optimized concentration of 2 × 10^4^ cells/well. Infection with *SL* (MOI10) was performed for 6 h. At the end of the infection, the media was replaced with XFp DMEM supplemented with glucose (10 mM), pyruvate (1 mM), and glutamine (2 mM) and incubated for 1 h in a non-CO2 incubator at 37 °C. The cartridge was hydrated with a Seahorse calibrant for 45–60 minutes in a non-CO2 incubator. Oligomycin (final concentration of 1.5 µM/well) and rotenone/antimycin (final concentration of 0.5 µM/well) (provided by the manufacturers) were loaded into ports A and B of the sensor cartridge and first loaded into the Agilent Seahorse XF HS Mini Analyzer (Agilent Technologies, Santa Clara, CA, USA) for calibration. Following calibration, the cell culture miniplate was loaded into the instrument to measure the oxygen consumption rate (OCR), extracellular acidification rate (ECAR), glycolytic ATP, mitochondrial ATP, total ATP, and ATP index according to the manufacturer's software. The levels of glycolytic and mitochondrial ATP after *SL* infection normalized to the level of ATP in the same cell lines without infection. The impact of ELMO1 on ATP production was assessed by comparing the level of ATP in C1 *SL*/C1 UN with that in E1 *SL*/E1 UN in both the glycolytic and the mitochondrial pathways. Agilent Seahorse Analytics software was used for data analysis.

### Seahorse XF cell Mito Stress test for the assessment of mitochondrial respiration

Seahorse analysis of mitochondrial respiration was performed via the Seahorse XFp Cell Mito Stress assay following the workflow provided by the manufacturer's instructions. C1 and E1 cells were plated and infected with *SL* as described above. The media was replaced with XFp DMEM supplemented with glucose (10 mM), pyruvate (1 mM), and glutamine (2 mM) and incubated for 1 h in a non-CO_2_ incubator at 37 °C. Following hydration of the cartridge with the Seahorse calibrant, the following inhibitors were injected into the cartridge according to the manufacturers' instructions: oligomycin (1.5 µM) in port A, FCCP (1.5  µM) in port B and rotenone/antimycin A (0.5 µM) in port C, followed by measurement of the OCR, ECAR, basal respiration, maximal respiration, spare respiratory capacity, and ATP production according to the manufacturer's software. Data analysis was performed with Agilent Seahorse Analytics software.

### Western blot

Cells were washed with phosphate-buffered saline (PBS) and lysed with radioimmunoprecipitation assay (RIPA) buffer. Mouse tissues were homogenized in RIPA buffer and then centrifuged to collect the cell lysates. The lysates were then separated by sodium dodecyl sulfate polyacrylamide gel electrophoresis (SDS/PAGE) using a 10% gel, transferred to a polyvinylidene difluoride (PVDF) membrane, blocked with 5% skim milk/phosphate-buffered saline (PBS) containing 0.1% Tween 20 for 1 h at room temperature (RT) and then incubated overnight at 4 °C with primary antibodies against the following targets: BolA Family Member 1/BOLA1 (Cat No 18017−1-AP, Proteintech, Rosemont, IL, USA), ELMO1 (B−7) (Cat No sc−271519, Santa Cruz Biotechnology, Santa Cruz, CA, USA) for C1/E1 cells, and ELMO1 (AB174298−1001, Abcam, Cambridge, United Kingdom) for mouse-derived samples; Dynamin-related protein 1/DRP1 (12957−1-AP- Proteintech), *p*-DRP1 (Cat No: 4494S, Cell Signaling Technology, Danvers, Massachusetts, USA), Sorting Nexin 5/SNX5 (Cat No sc−515215, Santa Cruz Biotechnology, Santa Cruz, CA, USA), Retinoic acid-inducible gene I/RIG−1 (Cat No sc−376845, Santa Cruz Biotechnology, Santa Cruz, CA, USA), Tubulin (Cat No 2144S, Cell Signaling Technology, Danvers, Massachusetts, USA), and GAPDH (Cat No Cell 2118S, Cell Signaling Technology, Danvers, Massachusetts, USA). Anti-mouse IgG, an HRP-linked antibody (Cat No 7076S, Cell Signaling Technology, Danvers, Massachusetts, USA), anti-rabbit IgG, and an HRP-linked antibody (Cat No 7074S, Cell Signaling Technology, Danvers, Massachusetts, USA) were used as secondary antibodies. At least 2−3 independent experiments were performed for each target. Band intensity was assessed using ImageJ and the results are presented as the mean ± SEM. *P values* were determined using unpaired Student’s t test and were considered significant if the values were <0.05.

### LDH assay

Supernatants were collected from uninfected and infected C1 and E1 cells and assayed via the LDH assay using LDH-Glo^TM^ Cytotoxicity Assay Kit (Promega, Madison, WI, USA) according to the manufacturer’s instructions as previously described.^19^ Briefly, supernatants were diluted, an equal volume of LDH Detection Reagent was added, and the luminescence was measured where culture media was used to subtract the background.

### Functional assay with DRP1-inhibitor Mdivi1 and ELISA of proinflammatory cytokine

C1 and E1 J774 macrophages (J774) were seeded at a cell density of 5 × 10^4^ cells/well in a 96-well plate. The following day, the cells were infected with *SL* at an MOI of 10 for 3 h or 6 h. One hour prior to infection, the cells were pretreated with 10 μg/ml Mdivi1 (Cat # 3982; Bio-Techne Corporation, Minneapolis, MN, USA). For the 6 h time point of infection, the extracellular bacteria were killed by gentamicin treatment (500 µg/mL) for 90 min, followed by treatment with low-dose gentamicin (50 µg/mL) for 3.5 h at 37 ^◦^C. Mdivi1 was added throughout the entire experiment. The cell supernatants were collected and processed for ELISA. TNF-*α* was measured using the mouse TNF-*α* DuoSet ELISA kit (Cat # DY410, R&D, Minneapolis, MN, USA) following the manufacturer’s instructions.

### Infection of mice

Age- and sex-matched WT and global ELMO1 KO C57BL/6 mice were infected with *SL1344* via oral gavage (5 × 10^7^ cfu/mouse) as described previously (Sayed et al., 2021). The mice were euthanized after 5 d of infection, and the ileum was collected for western blot analysis. The animals were bred, housed, used for all the experiments, and euthanized according to the University of California San Diego Institutional Animal Care and Use Committee (IACUC) policies under the animal protocol number S18086.

### Data repository

The dataset files were uploaded to Massive as a private dataset. The link to download them is as follows (ftp://MSV000091439@massive.ucsd.edu). The DOI for the dataset is as follows (doi:10.25345/C5M32NM0T), the massive identifier number is (MSV000091439), and the proteome exchange identifier number is (PXD040691).

### Statistics

The data reported in this study were analyzed using GraphPad Prism 10 (GraphPad Software Inc., San Diego, CA, USA)**.** The data are presented as the mean ± standard deviation (SD) or standard error of the mean (SEM); otherwise, they are specified. Analysis of the data was performed using Student’s t test or one-way ANOVA. *p* value < 0.05 was considered significant.

## Results

### The proteomic landscape of macrophages infected with *Salmonella*

**Figure 1.**
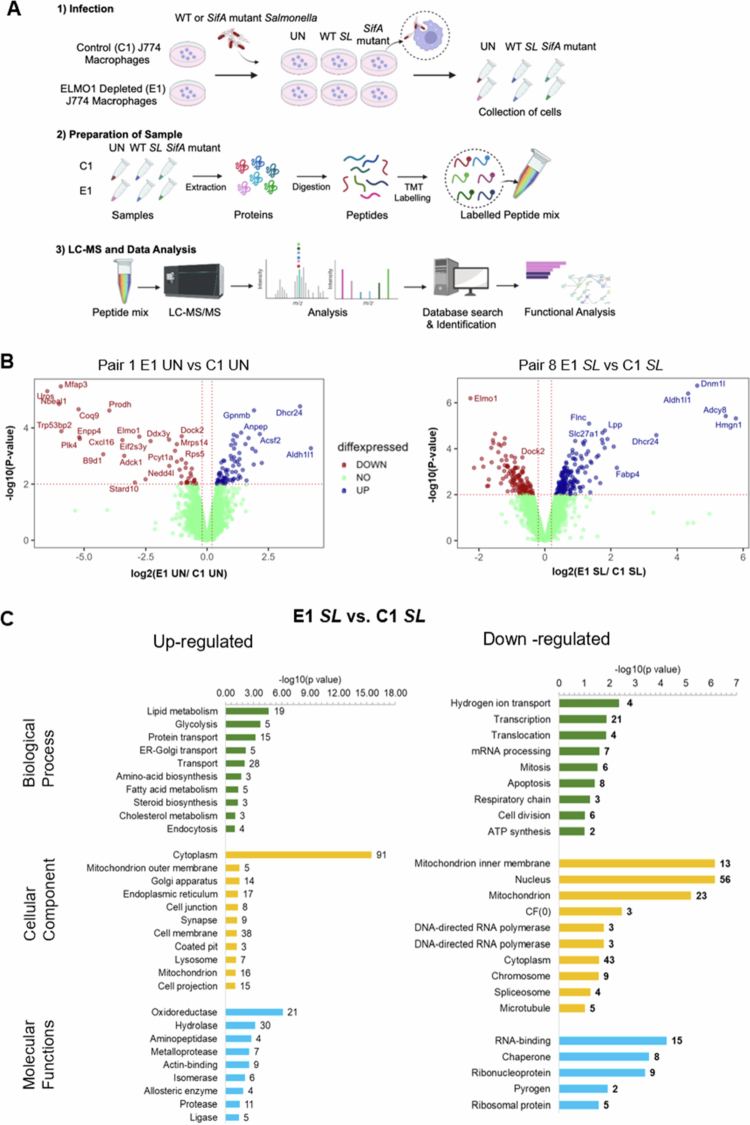
Proteomic profiling and Pathway Enrichment Analysis (PEA) of differentially expressed proteins in control and ELMO1-depleted murine macrophage J774 cells after infection with WT *Salmonella* (SL). A) Diagram showing the main steps in proteome profiling used in this study: (1) infection of control (C1) or ELMO1-depleted shRNA (E1) J774 macrophages with *Salmonella* enterica serovar typhimurium strain SL1344 (SL); (2) sample preparation, tandem mass tag (TMT) labeling of the pooled sample from the triplicate sets of each condition; (3) separation by liquid chromatography (LC) and mass spectrometry (MS) to quantify relative protein intensities by tags followed by analysis to predict the functions of the differentially expressed proteins. B) Volcano plots showing the statistical significance (log10 *p* value, Y axis) and fold change in protein expression (log2, X-axis) of different pairs, as indicated in the figures. (i) Comparison of host proteins of C1 (uninfected) vs host proteins of E1 (uninfected). (ii) Comparison of host proteins of C1 (SL) vs host proteins of E1 (SL). Red, blue, and green dots represent downregulated, upregulated, and no change in protein levels, respectively. C) GO enrichment and DAVID functional annotations for analysis of 293 proteins [upregulated proteins (*n* = 168) and downregulated proteins (*n* = 125)] in C1 SL vs E1 SL. The green bars represent biological functions, the yellow bars represent cellular components, and the blue bars represent molecular functions. The left panel represents upregulated proteins, and the right panel represents downregulated proteins.

To understand the major host pathways linked to ELMO1 after *Salmonella* infection, murine macrophage J774 cells (either control-C1 or ELMO1-depleted E1 cells) were infected with WT *Salmonella* (*SL*), *SifA mutan*t *SL* and compared with the uninfected controls (Figure 1A). The proteomics identified a total of 7777 proteins with 200148 peptide spectral matches in the six conditions with C1 UN, E1 UN, C1 *SL*, E1 *SL*, C1 *SifA*, and E1 *SifA* (Figure 1A, Figure S1), with a false discovery rate of 1%. First, we performed quantitative proteomics and identified the altered proteins in the host cells of uninfected control cells (C1 UN and E1 UN) and cells infected with *SL* (C1 *SL* and EL *SL*) (Figure 1B). The volcano plot shows the deregulated proteins for both statistical significance and fold change. Volcano plots were constructed to identify the most biologically significant proteins (with greater fold changes in expression) among the pairs mentioned inFigure 1B. We compared a total of 10 pairs, as shown inTable 1. In the pairs P1 (E1 UN vs C1 UN), P8 (E1 *SL* vs C1 *SL*), P7 (C1*SifA* vs C1 *SL*), P4 (E1 *SifA* vs E1 *SL*), P9 (E1 *SifA* vs. C1 *SifA)* and P10 (E1 *SifA* vs C1 *SL*) 74, 168, 2, 5, 60 and 86 significantly upregulated proteins (blue dots) and 40, 126, 19, 29, 37 and 61 significantly downregulated proteins (red dots) were identified, respectively (Tables S1--S10). In all E1 cell conditions, the ELMO1 protein level was lower than that in C1 cells, which confirmed the confidence of the multiplex proteomics technology and confirmed the characteristics of these ELMO1-depleted macrophages (E1 cells) (Figure 1B).

**Table 1.** List of sample comparisons.

Pair	Comparison
1	E1 UN vs. C1 UN
2	E1 SL vs. E1 UN
3	E1 *SifA* mutant vs. E1 UN
4	E1 *SifA* vs. E1 SL mutant
5	C1 SL vs. C1 UN
6	C1 *SifA* mutant vs. C1 UN
7	C1 *SifA* vs. C1 SL mutant
8	E1 SL vs. C1 SL
9	E1 *SifA* mutant vs. C1 *SifA* mutant
10	E1 *SifA* vs. C1 SL mutant

Furthermore, the differentially expressed proteins (DEPs) in pair 1 E1 UN vs C1 UN were analyzed via the Gene Ontology (GO) enrichment pathway (GO) using the **D**atabase for **A**nnotation, **V**isualization and **I**ntegrated **D**iscovery (DAVID) version 2021. Importantly, the transforming growth factor beta receptor signaling pathway (TGF) and Rac protein signal transduction were among the enriched biological processes associated with the downregulated proteins (Figure S2). This finding is not surprising since ELMO1 interacts with DOCK180 and is involved in Rac activation^38^. Interestingly, the upregulated proteins were associated with lipid metabolic processes, vesicle-mediated and intracellular protein transport, and filament organization and assembly (Figure 1C, Figure S2). These findings align with previous findings showing that ELMO1 plays a role in the filament assembly and endocytosis/engulfment of bacteria.^18^^,^^39^^,^^40^

After infection with *SL*, functional annotation of significantly upregulated proteins from E1 *SL* vs C1 *SL* identified major proteins linked with lipid metabolism, transport, apoptosis, and ATP synthesis (Figure 1C) while transcription, apoptosis, and hydrogen ion transport were some of the major functions associated with downregulated proteins (Figure 1C). Importantly, the protein‒protein interaction (PPI) networks for DEPs revealed many pathways linked to mitochondrial activity, such as oxidative phosphorylation, metabolic pathways, energy metabolism, chemokine signaling pathways, and inositol phosphate metabolism (Figure S3).

### Alteration of mitochondrial functions in ELMO1-depleted macrophages compared to control during *Salmonella* infection

**Figure 2.**
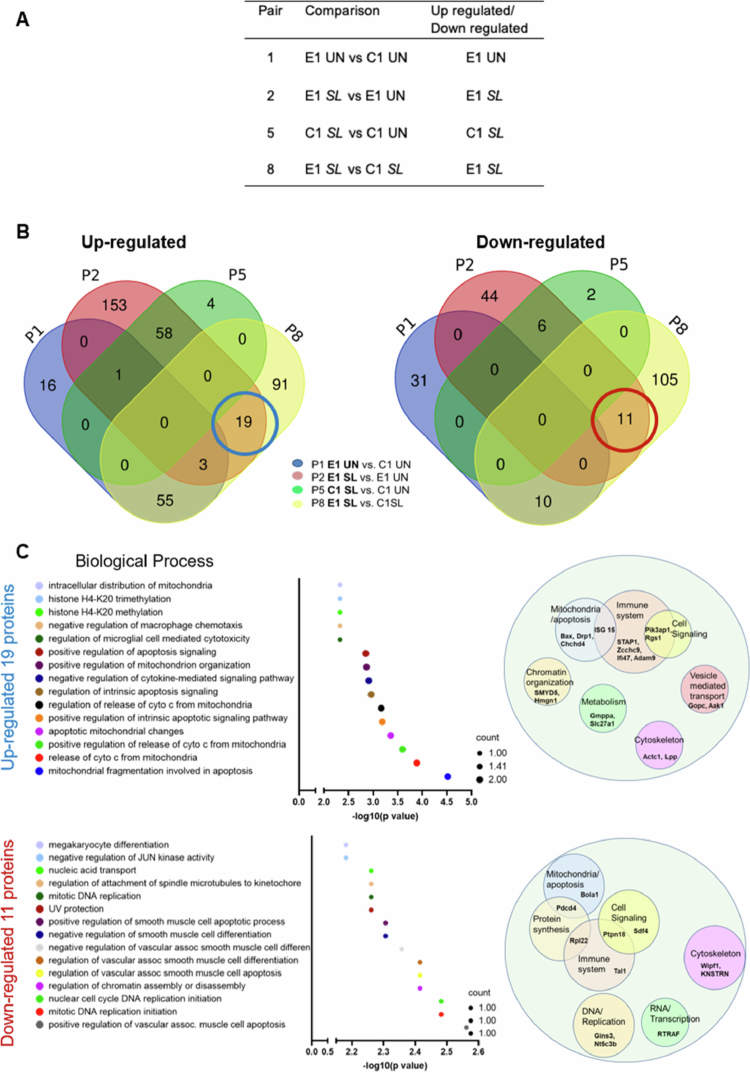
ELMO1 regulates the pathways involved in mitochondrial function, vesicular transport, and host immune responses following WT *Salmonella* infection. A) Table showing the pairs and the respective proteomic datasets considered for the comparisons: pair 1 (E1 un versus C1 un), pair 2 (E1 SL versus E1 un), pair 5 (C1 SL versus C1 un), and pair 8 (E1 SL versus C1 SL). B) Venn diagrams showing the comparison of significantly upregulated proteins (Left) and downregulated proteins (Right) from the above-mentioned pairs. C) (left panel) GO term enrichment analysis showing the biological processes of the 19 upregulated [circled “blue” in the Venn diagram (B)] and 11 downregulated proteins [circled “red” in the Venn diagram (B)] unique to the E1-SL conditions (between P2 and P8, where E1 SL is compared with E1 un and C1 SL). The size of the circle in the graph corresponds to the number of proteins in each pathway. The right panel shows the major functions associated with these proteins by using PubMed and UniProt search (last accessed on 15 December 2023). The size of the circles is arbitrary depending on the number of proteins associated with the same function.

To identify the proteins involved with ELMO1 during *SL* infection, we plotted a Venn diagram of upregulated and downregulated proteins from pairs 1, 2, 5, and 8, where C1 and E1 cells either uninfected or infected with *SL* were compared (Figure 2A). A comparison of DEPs from pairs 1, 2, 5, and 8 revealed that 153 (pair 2), 91 (pair 8), and 19 (common to pairs 2 and 8) upregulated unique proteins in E1 cells after *SL* infection. In addition, 44 (pair 2), 105 (pair 8), and 11 (common to pair 2 and pair 8) proteins were downregulated in E1 cells during *SL* infection (Figure 2B). Although 153 (pair 2 upregulated) and 44 (pair 2 downregulated) proteins were identified as DEPs, these proteins were compared to those in E1 uninfected individuals. Since we were interested in DEPs in E1 cells compared to C1 cells during *SL* infection, we further evaluated 19 upregulated proteins and 11 downregulated proteins that were common to both pair 2 and pair 8 (Figure 2B). GO enrichment analysis using Enrichr revealed that the 19 upregulated and the 11 downregulated proteins are involved in various functions predominantly linked with the immune response, cell signaling, and the mitochondrial pathway. Other cellular functions associated with the upregulated proteins included vesicle-mediated transport, metabolism, and chromatic organization (Figure 2C upper right panel,Table 2) while protein synthesis, DNA replication, and transcription were enriched among the downregulated proteins (Figure 2C lower right panel,Table 3).

**Table 2.** Functions of 19 upregulated proteins identified in E1-SL conditions.

Uniprot ID	Gene name	Count	Fold change		*p*value		
			E1SL vs E1UN	E1SL vs C1SL	E1SL VS E1UN	E1SL vs C1SL	Function
Q9JL25 (Regulator of G-protein signaling 1)	Rgs1	4	0.970418	0.897749044	0.001341	0.004249267	Controls G protein-coupled receptor signaling cascades
Q9JM90 (Signal-transducing adaptor protein 1)	Stap1	25	0.660728	0.553939614	0.001504	0.010586904	Acts as an adapter molecule, downstream of KIT with a role in proliferation or differentiation of hematopoietic stem cells.
Q64339 (Ubiquitin-like protein ISG15)	Isg15	7	1.148553	0.993042728	0.001271	0.000775434	Essential role in the innate immune response during viral infection
Q3TYX3 (Histone-lysine *N*-trimethyltransferase SMYD5)	Smyd5	14	1.129451	1.094277896	9.05E−05	0.000438635	Specifically, trimethylates 'Lys−20' of histone H4, resulting epigenetic transcriptional repression
Q8K1M6 (Dynamin−1-like protein)	Dnm1l	4	4.788223	4.606902005	1.43E−06	4.61738E−07	Role in mitochondrial and peroxisomal division, involved in SCV division
Q8BFW7 (Lipoma-preferred partner homolog)	Lpp	8	1.857145	1.81965913	1.35E−05	2.5934E−05	functions at sites of cell adhesion and maintians cell shape and motility
Q9EQ32 (Phosphoinositide 3-kinase adapter protein 1)	Pik3ap1	47	0.489788	0.428070683	0.004343	0.01393964	Role in B-cell development.
P18608 (Non-histone chromosomal protein HMG−14)	Hmgn1	3	5.959335	5.778013922	0.000517	2.6245E−05	Binds nucleosomal DNA, altering the interaction between the DNA and the histone octamer
Q922H4 (Mannose−1-phosphate guanyltransferase alpha)	Gmppa	14	0.644279	1.000269028	0.011173	0.0021276	homolog of GMPPB, role in allosteric feedback inhibition of GMPPB by GDP-mannose
Q8R1J3 (Zinc finger CCHC domain-containing protein 9)	Zcchc9	5	2.144209	1.756436843	2.86E−05	6.23049E−05	Regulates NFkB and serum response element
Q60714 (Long-chain fatty acid transport protein 1)	Slc27a1	6	1.480474	1.746611823	0.000105	1.54549E−05	Facilitates the import of long-chain fatty acids (LCFA) into the cell at the plasma membrane
Q07813 (Apoptosis regulator BAX)	Bax	33	1.660258	1.623629771	0.003187	0.000827434	Binds BCL2 or E1B 19k, accelerates programed cell death
Q8VEA4 (Mitochondrial intermembrane space import and assembly protein 40)	Chchd4	20	1.412926	1.083300978	0.007354	0.014548325	Key component of a redox-sensitive mitochondrial intermembrane space import machinery, regulates biogenesis of respiratory chain complexes
P68033 (Actin, alpha cardiac muscle 1)	Actc1	11	0.767347	0.677395837	0.004145	0.004390421	Role in various types of cell motility
A0A571BEI2 (AP2 associated kinase 1)	Aak1	34	1.137022	1.001130207	0.00658	0.007279068	involved in clathrin mediated endocytosis
K3W4Q9 (Golgi associated PDZ and coiled-coil motif containing)	Gopc	16	1.19688	1.123943904	0.000216	0.000689496	Acid-sensing ion channel 3
A0A140LHU0 (A disintegrin and metallopeptidase domain 9 (meltrin gamma))	Adam9	21	0.625194	0.956965521	0.002315	0.000488973	Involved in developmental process, inflammation, viral entry and degenerative diseases (PMID: 33096780)
F8VQ05 (FRY like transcription coactivator)	Fryl	19	0.538097	0.700915495	0.005102	0.001925933	Unknown function
Q61635 (GTP-binding protein)	Ifi47	32	0.375518	0.569951335	0.019068	0.008292658	Interferon gamma inducible protein 47 The hnRNPU KO of Raw264.7 macrophages after SL infection identified Ifi47 (PMID: 34305890)

**Table 3.** Functions of 11 downregulated proteins identified in E1-SL conditions.

Uniprot ID	Gene Name	Count	Fold Change		pValue		
			E1SL vs E1UN	E1SL vs C1SL	E1SL vs E1UN	E1SL vs C1SL	Function
Q8K1I7 (WAS/WASL-interacting protein family)	Wipf1	23	−0.87827	−0.745782801	0.00084	0.003143987	Involved in the reorganization of the actin cytoskeleton, filopodia formation, and cell ruffles through Rho family GTPases
P67984 (50S ribosomal protein L22)	Rpl22	39	−0.61289	−0.973157854	0.005435	0.005121823	Component of the large ribosomal subunit.
Q9CQE8 (RNA transcription, translation, and transport factor)	RTRAF	42	−0.58507	−0.796599585	0.017195	0.004650795	Regulate protein kinase activity and RNA polymerase II complex binding activity. It acts as tRNA splicing factor
Q9D8S9 bolA-like 1 (E. coli)	Bola1	35	−0.37144	−0.549684534	0.01896	0.006088123	Acts as a mitochondrial iron-sulfur (Fe-S) cluster assembly factor Affects fitness and enhances *Salmonella enterica* Serovar Typhimurium virulence. Modulates of the metabolic processes and regulates the stress resistance and virulence during *Salmonella* infection
Q61823 (programmed cell death 4)	Pdcd4	7	−1.12737	−0.784102085	0.001091	0.004510785	Inhibits protein synthesis by interaction between EIF4A1. Involved in apoptosis and acts as tumor suppressor protein.
Q61112 stromal cell derived factor 4	Sdf4	14	−0.56546	−0.601593152	0.003838	0.005495372	Regulation of calcium-dependent activities in the endoplasmic reticulum lumen or post-ER compartment.
Q9D9Z1 (kinetochore localized astrin (SPAG5) binding protein)	KNSTRN	6	−0.92195	−0.879363232	0.003398	0.003381794	The astrin (SPAG5)-kinastrin (SKAP) complex contributes to stable microtubule-kinetochore attachments, plays a role in mitosis
Q9CY94 GINS complex subunit 3	Gins3	16	−0.51269	−0.895096802	0.013036	0.0013205	Plays a role in DNA replication, and progression of DNA replication forks.
Q3UFY7 (7-methylguanosine phosphate-specific 5’-nucleotidase)	Nt5c3b	3	−0.75666	−1.2173592	0.003031	0.000147499	Role in protection of cells against undesired salvage of m7GMP and its incorporation into nucleic acids
Q61152 (Tyrosine-protein phosphatase non-receptor type 18)	Ptpn18	11	−0.60059	−0.783574071	0.010004	0.002148645	Contribute to growth and differentiation of hematopoietic cells.
P22091 (T-cell acute lymphocytic leukemia protein 1)	**Tal1**	9	−0.63524	−0.628314456	0.006238	0.005025647	Involved in hemopoietic differentiation, and regulation of erythroid differentiation.

### ELMO1 affects glycolysis and oxidative phosphorylation following *Salmonella* infection

**Figure 3.**
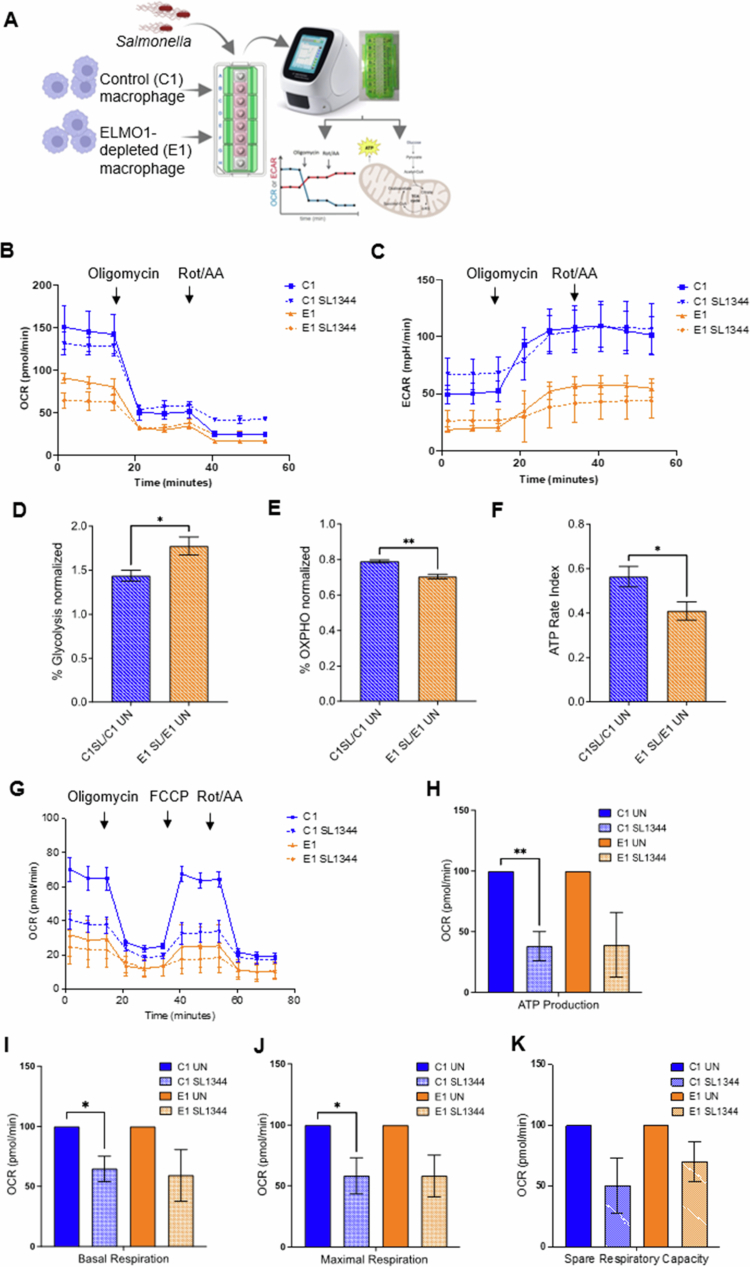
The functional role of ELMO1 in mitochondrial dynamics following infection with WT *Salmonella* by bioenergetic analysis and ATP production. A) Schematic representation of the experimental workflow for the Seahorse assays. Control shRNA J774 cells (C1) and ELMO1-shRNA J774 (E1) cells were seeded in XF HS Miniplate and challenged with the WT SL1344 strain (SL) or left untreated. After 6 h, the plate was prepared for the ATP rate or Mito Stress assay, and the assay was run using the Agilent Seahorse XF HS Mini Analyzer. B) Representative kinetic profile of oxygen consumption rate (OCR) measurements (pmol/min) presented as averages of three individual replicates per condition ± standard deviations in C1 and E1 at the basal level or following 6 h of infection with *Salmonella* (SL). The data are presented as averages of three technical replicates. C) Representative kinetic profile of the extracellular acidification rate (ECAR) (mpH/min) in C1 and E1 at the basal level or following 6 h of infection with SL. The assay was repeated three times and was performed in triplicates using the Seahorse Xfp real-time ATP rate assay. The data are presented as averages of three technical replicates. D-E) Quantification of the percentage of glycolysis (D). Oxidative phosphorylation (E) in C1 and E1 following SL infection for 6 h, as shown in B and C. Values were normalized to the respective values in uninfected C1 and E1. F) Quantification of the ATP rate index (ratio of mitochondrial ATP to glycolytic ATP production) in C1 and E1 following SL infection for 6 h as described in B, C, and D. Values were normalized to the respective C1 E1 untreated values. G) Representative kinetic profile of the oxygen consumption rate (OCR) (pmol/min) of the Seahorse XF Cell Mito Stress Test in C1 and E1 cells at the basal level or following 6 h of infection with SL. The data are presented as averages of three technical replicates. H-K) The OCR of (H) ATP production, (I) basal respiration, (J) maximal respiration, and (K) spare respiratory capacity of the experiments in G were determined. The data are presented as averages of three independent experiments, where * indicates a *p* value ≤ 0.05 and ** indicates a *p* value ≤ 0.01, as determined by two-tailed Student's t test.

To investigate whether ELMO1 affects host metabolism during *Salmonella* infection, C1 and E1 cells were infected with *SL* for 6 h, followed by measurement of the oxygen consumption rate (OCR) and the extracellular acidification rate (ECAR), as depicted inFigure 3 A‒C, using the Seahorse XFp real-time ATP rate assay. We found that *SL* infection induces a significant increase in glycolysis and a significant decrease in oxidative phosphorylation (OXPHOS) (Figure 3 B-C, Figure S4), indicating a metabolic shift in macrophages from OXPHOS to glycolysis upon *SL* infection, a phenomenon previously observed in other studies.^41-46^ Importantly, we observed that the changes induced by *SL* are exacerbated by the depletion of ELMO1, as reflected by the increased percentage of glycolysis (Figure 3D) and a further reduction in the percentage of oxidative phosphorylation (Figure 3E) in E1 cells compared to C1 macrophages during *SL* infection. Moreover, our results revealed a reduction in the ATP rate index, which represents the ratio of mitochondrial ATP to glycolytic ATP production, in E1 cells following *SL* infection (Figure 3F, Figure S4). Next, we performed a Seahorse XFp Cell Mito Stress assay to determine the impact of ELMO1 on aerobic respiration. We confirmed a reduction in the OCR, indicating an overall decrease in mitochondrial respiration (Figure 3G) during infection. The calculated values derived from the Seahorse XFp analyzer demonstrated a significant decrease in mitochondrial ATP production (Figure 3H), basal respiration (Figure 3I), maximal respiration (Figure 3J), and spare respiratory capacity (Figure 3K) in C1 and E1 cells following *SL* infection. To assess cell health under each of these conditions, we used the LDH assay for measuring cytotoxicity.^19^ C1-E1 cells following infection did not exhibit any remarkable cell death (Figure S5).

### Validation of DEPs in ELMO1-depleted macrophages after *Salmonella* infection

**Figure 4.**
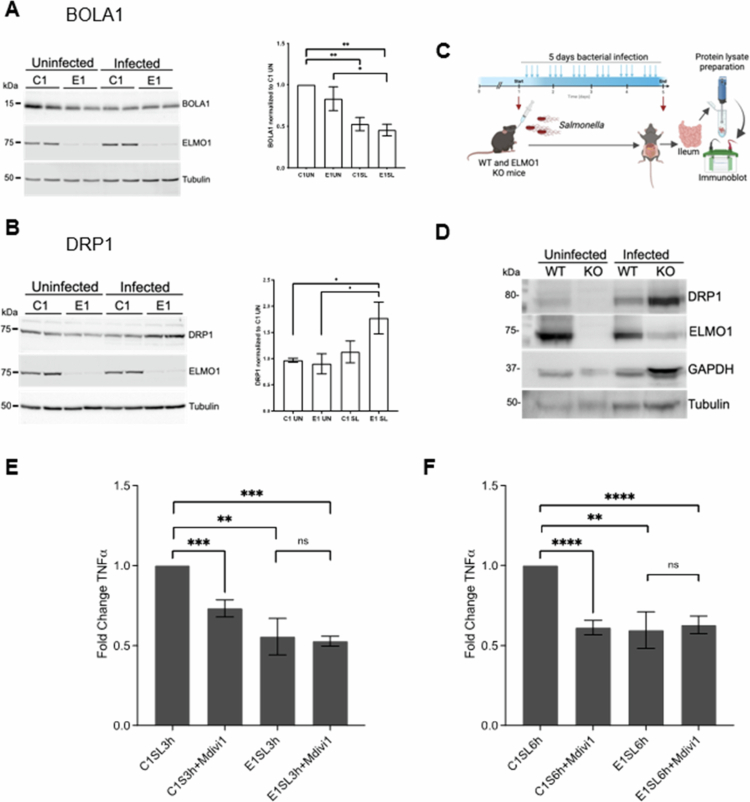
Validation of the targets via western blotting and functional analysis. A-B Western blot of BOLA1 (A) and DRP1 (B) in C1-E1 cells following infection with SL. The densitometry was performed using three independent experiments where * indicates *p* value ≤ 0.05 and ** indicates *p* value ≤ 0.01 as assayed by two-tailed Student's t-test. C. Schematic illustration of the results of the animal experiments used to assess the expression of DRP1 shown in D. D. Western blot analysis of DRP1 in tissue samples from the ileum of WT and ELMO1 KO mice after 5 d of SL infection; the results are presented in C. E‒F. TNF-*α* cytokine levels in the supernatant of C1 and E1 cells after 3 h (E) and 6 h (F) of infection with SL in the presence of the DRP1 inhibitor mDivi−1 were measured via ELISA. The data represent the fold change (mean ± SEM) compared to C1 from the values collected from two separate experiments with four biological replicates. * Indicates p ≤ 0.05, ** indicates p ≤ 0.01, *** indicates p ≤ 0.001, **** indicates p ≤ 0.0001 as assayed by two-tailed Student's t test.

To validate the proteomics data and the effect of ELMO1 on the identified proteins, we performed *in vitro* and *in vivo* infection experiments and assessed the expression of selected proteins via western blotting. We selected one downregulated protein, BolA family member 1 (BOLA1), and one upregulated protein, dynamin-related protein 1 (DRP1), from the selected list based on their involvement in mitochondrial function, fold change and *p* values. Among the 11 downregulated proteins, BOLA1 is a mitochondrial protein that affects mitochondrial morphology, interacts with the mitochondrial monothiol glutaredoxin GLRX5, protects cells from oxidative stress, and influences other mitochondrial processes.^47^^,^^48^ *In vitro* experimental data revealed that the expression levels of BOLA1 decreased after *SL* infection and were significantly lower in E1 *SL*compared to all uninfected cells (Figure 4A).

On the other hand, DRP1, which is a member of the dynamin superfamily of GTPases involved in mitochondrial fission^49^^,^^50^, was found to be upregulated in E1 *SL* compared to other conditions. We noticed that the expression of DRP1 was significantly upregulated in E1 *SL* compared to all the uninfected samples as well as in C1 *SL* (Figure 4B). To further confirm our findings, we assessed the level of DRP1 in the ileum of wild-type and ELMO1-KO mice infected with *SL* for 5 d (Figure 4C). Interestingly, the highest level of DRP1 was detected in the ileum of *SL*-infected ELMO1-KO mice (Figure 4D). In contrast, the downregulated protein BOLA1 is indeed expressed at lower levels in ELMO1 KO ileum (Figure S6 A).

To understand the mechanism of ELMO1-mediated mitochondrial fission by DRP1, we assessed the level of pDRP1, as pDRP1 is recruited onto the outer mitochondrial membrane (OMM) and regulates fission.^51^ We found that the level of pDRP1 increased after 1 h of *SL* infection and was higher in E1SL compared to C1SL (Figure S6B).

### Functional analysis of DRP1 in inflammation

Previous studies have shown that DRP1 modulates the production of inflammatory cytokines in response to immune stimulation and infection.^52^^,^^53^ To understand whether the role of ELMO1 is linked to the function of DRP1, we used the DRP1 inhibitor Mdivi−1^54^ in C1 and E1 cells following infection with *SL* for 3 h (Figure 4E) and 6 h (Figure 4F). The level of TNF-*α* was lower in E1 compared to C1 before Mdivi−1 treatment. TNF-*α* levels were significantly reduced in *SL*-infected C1 cells following Mdivi−1 treatment but did not decrease further in E1 cells after infection (Figure 4E and F, and Supplement Figure S5). This finding suggests that the majority of the DRP1-dependent TNF-*α* response is mediated by ELMO1.

### Pathway enrichment analysis of DEPs controlled by ELMO1 after infection with *SifA* mutant

**Figure 5.**
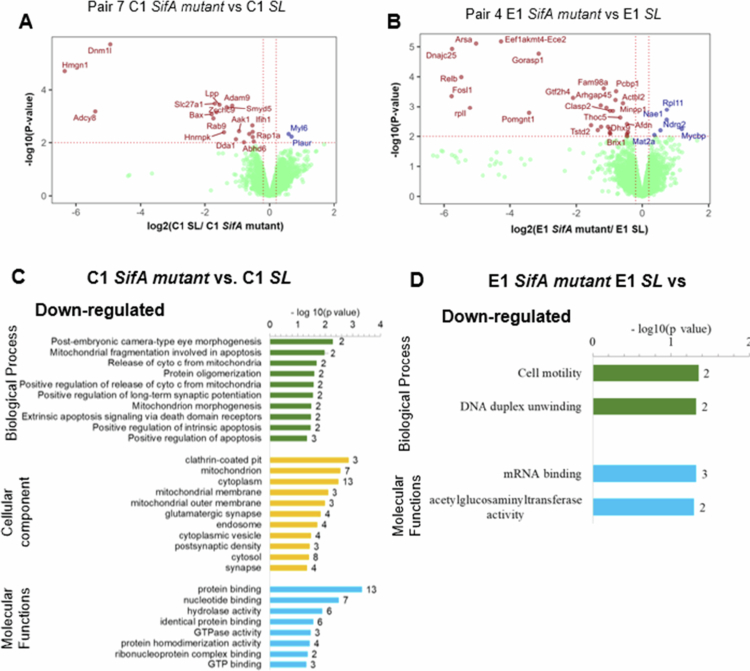
PEA and DAVID analysis for control shRNA (C1) and ELMO1 shRNA (E1) in J744 cells after infection with the SifA mutant strain. A & Hamp; C. Volcano plots showing the statistical significance (log10 *p* value, Y axis) and fold change in protein expression (log2, X-axis) of different pairs, as indicated in the figures. Pair 7 C1 SifA mutant vs C1 SL in A and Pair 4 E1 SifA mutant vs E1 SL in C. Red, blue, and green dots represent downregulated, upregulated, and no change in protein levels, respectively. B & Hamp; D. GO term enrichment analysis and DAVID functional annotations for DEPs related to biological functions (green), cellular components (yellow), and molecular functions (blue) of downregulated proteins of Pair 7 (C1 SifA mutant vs C1 SL) in B and downregulated proteins of Pair 4 (E1 SifA mutant vs E1 SL) in D.

Previously, we showed that ELMO1 interacts with the *Salmonella* effector SifA.^24^ Here, we determined the DEPs after *Salmonella* effector protein SifA during infection in the presence or absence of ELMO1. We performed an analysis of the proteins in pair 7 (C1 *SifA* vs C1 *SL*), pair 4 (E1 *SifA* vs E1 *SL*)*,* pair 9 (E1 *SifA* vs C1 *SifA*), and pair 10 (E1 *SifA* vs C1 *SL*). A comparison of proteins from pair 7 revealed 19 downregulated proteins and 2 upregulated proteins, myosin light chain peptide 6 (Myl6) and plasminogen activator urokinase receptor PLAUR (Figure 5A). The two upregulated proteins are involved in cell proliferation, migration, and apoptosis (PLAUR) or the motor protein myosin (My16). Similarly, a comparison of pair four proteins revealed 30 and 5 proteins with elevated expression levels in the case of *SL* and SifA mutant infection, respectively (Figure 5B). The five upregulated proteins are involved in cellular processes (Rpl11, NudE, and Mat2a) and are linked to Myc pathways (Ndrg2 and Mycbp).

GO analysis of the downregulated proteins in C1 *SifA* vs C1 *SL* from pair 7 (Figure 5A) highlighted pathways involved in mitochondrial functions and apoptosis, protein oligomerization, and embryonic development functions (Figure 5C). GO analyses of proteins in E1 *SifA* vs E1 *SL* in pair 4 (Figure 5B) revealed that cell motility and DNA duplex unwinding are significantly associated with downregulated E1 proteins during *SifA* infection (Figure 5D).

**Figure 6.**
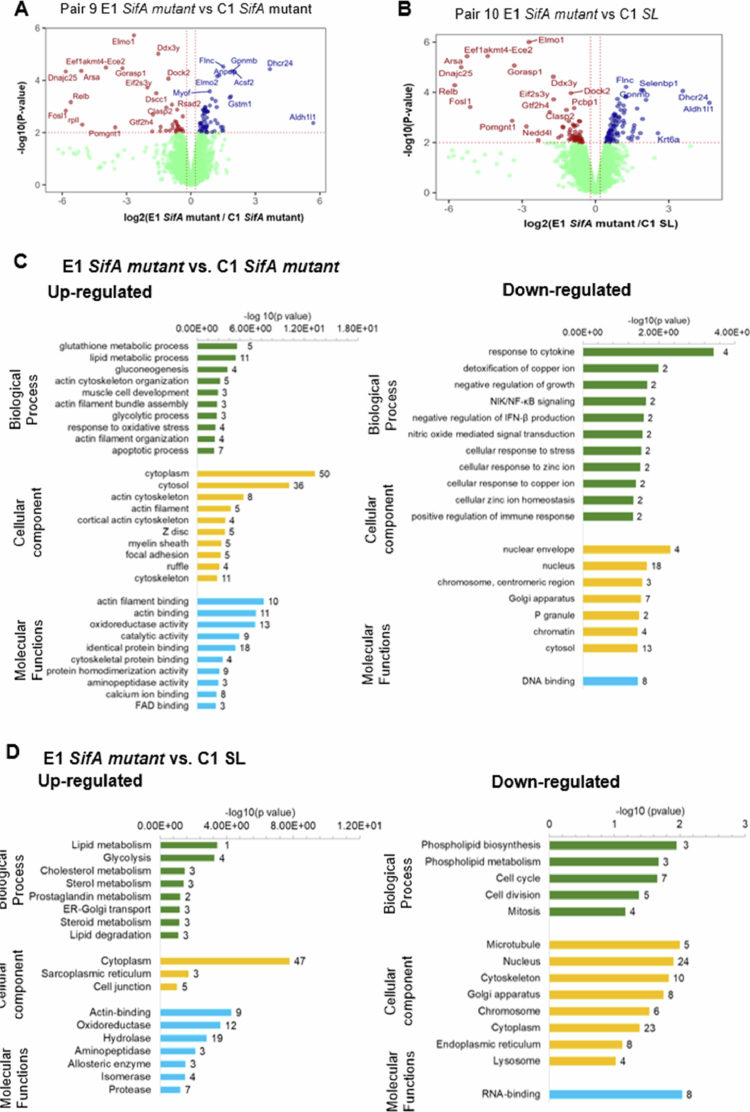
PEA and DAVID analysis for control shRNA (C1) and ELMO1 shRNA (E1) J744 cells after infection with the WT SL and SifA mutant strains. A & Hamp; C. Volcano plots showing the statistical significance (log10 *p* value, Y axis) and fold change in protein expression (log2, X-axis) of different pairs, as indicated in the figures. Pair 9 E1 SifA mutant vs C1 SifA mutant in A and Pair 10 E1 SifA mutant vs C1 SL in C. B & D. GO term enrichment analysis and DAVID functional annotations for DEPs of biological functions (green), cellular component (yellow), and molecular functions (blue) of (B) upregulated proteins (left) and downregulated proteins (right) of Pair 9 E1 SifA mutant vs C1 SifA mutant) and (D) upregulated proteins (left) and downregulated proteins (right) of Pair 10 (C1 SL vs E1 SifA mutant).

Next, we checked the effects of ELMO1 and SifA in pair 9 *SifA mutant* infection (Figure 6A) of C1 and E1 cells and in pair 10 with E1 *SifA* vs C1 *SL* (Figure 6B). The proteins shown inFigure 6A that are linked with metabolic functions such as glucose, lipids, and cholesterol were expressed at higher levels in E1 cells compared to C1 cells, whereas proteins involved in the immune response, cytokine response mediated by NFĸB and stress response presented lower expression levels (Figure 6C). Similarly, a comparison of pair 10 with E1 *SifA* vs C1 *SL* (Figure 6B) revealed 86 proteins with relatively high levels in E1 after SifA mutant infection. Functions associated with metabolism (lipid, glycolysis, and sterol) and ER‒Golgi transport were the most enriched biological processes (Figure 6D). Additionally, 61 proteins presented relatively high expression levels in C1 cells infected with *SL,* and the cell cycle and mitosis were the prominent biological processes identified via enrichment analysis (Figure 6D). Among the various pathways and functions that are controlled by ELMO1 during *SL* and *SifA mutan*t infection, most of these pathways are associated with metabolism and host immune/defense responses.

### The differentially regulated proteins controlled by ELMO1-SifA interaction

**Figure 7.**
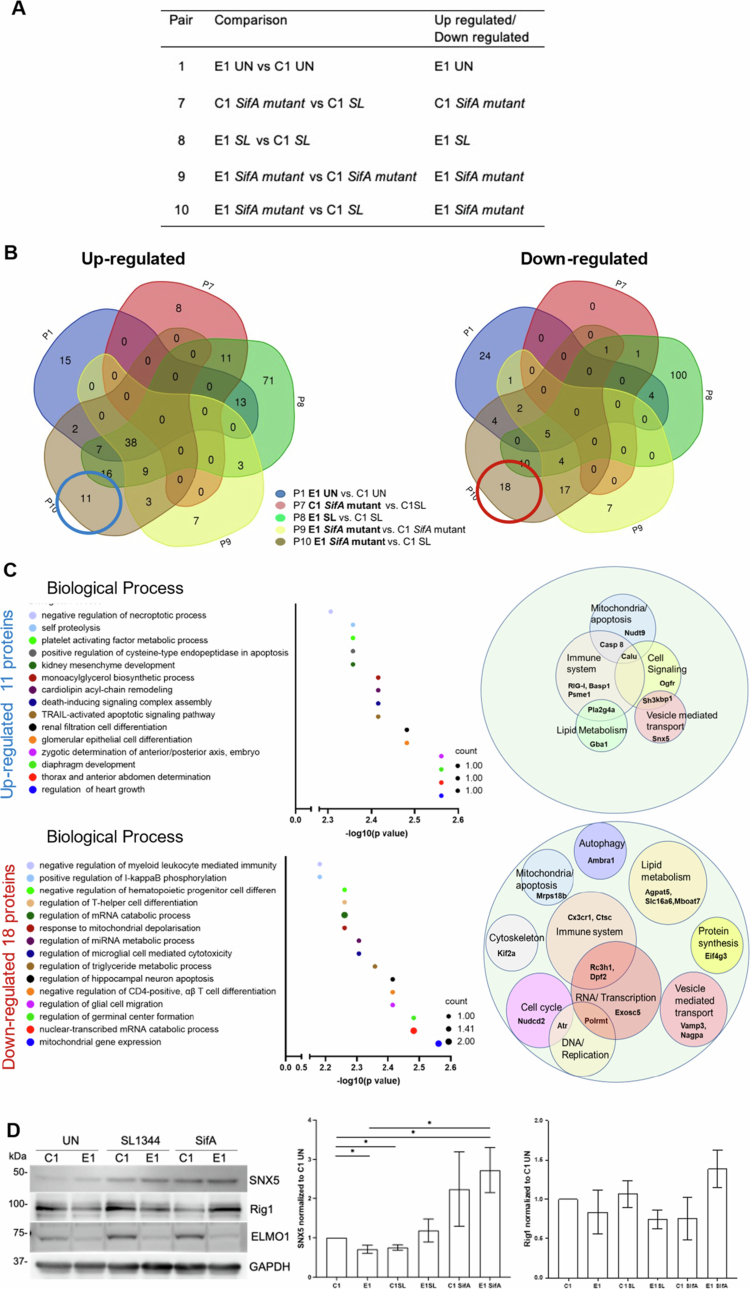
The differentially expressed protein profiles are regulated by the host ELMO1 and Salmonella effector SifA. A. Table showing the pairs and the respective proteomic datasets considered for the comparison: Pair 1 (C1 Un vs E1 Un), Pair 7 (C1 SL vs C1 SifA mutant), Pair 8 (C1 SL vs E1 SL), Pair 9 (C1 SifA mutant vs E1 SifA mutant), and Pair 10 (C1 SL vs E1 SifA mutant). B. Venn diagram comparing significantly upregulated proteins (Left) and downregulated proteins (Right) from the above-mentioned pairs in (A). C. (left) GO term enrichment analysis for biological processes of DEPs in E1 SifA compared to other conditions in the above-mentioned pairs. Eleven upregulated proteins (upper panel circled blue in Venn diagram B) and 18 downregulated proteins (lower panel circled red in Venn diagram B) were identified. The size of the circle in the graph corresponds to the number of proteins in each pathway. (Right) Represents the major functions associated with these proteins according to a PubMed and UniProt search (last accessed on Jan 15, 2023). The size of the circles is arbitrary depending on the number of proteins associated with the same function. D) Western blot analysis of the vesicle transport protein SNX5 and the immune system protein RIG1 in C1-E1 cells following infection with SL. Densitometry was performed using three independent experiments where * indicates *p* < 0.5 as determined by two-tailed Student's t test.

To identify the proteins involved in *SifA* infection in the presence and absence of ELMO1, we plotted a Venn diagram of upregulated and downregulated proteins from the following pairs: pair 1 (E1 Un vs C1 un), pair 7 (C1 *SifA* vs C1 *SL*), pair 8 (E1 *SL* vs C1 *SL*), pair 9 (*E1 SifA vs* C1 *SifA)*, and pair 10 (*E1 SifA* vs C1 *SL)* (Figure 7A). Accordingly, 11 proteins with higher expression levels and 18 proteins with lower expression levels were found to be unique to E1 infected with the *SifA* mutant (Figure 7B).

The immune response, mitochondrial functions/apoptosis, lipid metabolism, and vesicle-mediated transport terms were the most enriched GO terms associated with both the upregulated and the downregulated proteins (Figure 7C,Table 4, andTable 5). Other enriched pathways associated with the downregulated targets included autophagy, DNA replication, and transcription (Figure 7C andTable 5). As the immune response and vesicle-mediated transport were enriched pathways enriched in both upregulated and downregulated proteins, we validated the proteomics findings by focusing on two targets from these pathways: SNX5 and RIG1. SNX5, known as sorting nexin 5, plays a role in endosomal trafficking via interaction with the retromer complex (VPS35, VPS26, and VPS29).^55^ RIG-I (retinoic acid-inducible gene-I) is a cytosolic viral RNA sensor with a role in the activation of innate immunity.^56^ The protein level of SNX5 was increased in E1 cells after *SL* and *SifA* infection, and the highest level was detected in *SifA* mutant-infected E1 cells (Figure 7D). Similarly, *SifA* mutant-infected E1 cells presented the highest expression of RIG−1 (Figure 7D).

**Table 4.** Functions of 11 upregulated unique proteins to E1-SifA conditions.*SifA* conditions.

Uniprot ID/protein	Gene	Count	Fold change	pValue	Function
Q6XLQ8 (Calumenin)	Calu	52	0.861832837	0.01268679	Contributes to ER-Ca2 + homeostasis Calumenin is developed during proteomics analysis of *Salmonella* Typhimurium infection
Q6Q899 (Orthologous to human DDX58 (DExD/H-box helicase 58))	Rigi RNA sensor RIG-I	92	0.624993687	0.035331374	RIG-I, together with RNA polymerase III, senses viral DNA. RIG-I detects the mRNA of *Salmonella*Typhimurium in nonimmune cells
P47713 (Cytosolic phospholipase A2. (PLA2))	Pla2g4a	48	0.469264737	0.038163132	Plays a role in allergic reactions, parturition, fertility, neuronal death, membrane lipid remodeling and production of inflammatory mediators.
Q8BVU5 (ADP-ribose pyrophosphatase, mitochondrial)	Nudt9	31	0.560039044	0.024352214	Hydrolysis of ADP-ribose to AMP and ribose 5’-phosphate.
Q6P7W2 (SH3 domain-containing kinase-binding)	Sh3kbp1	3	-0.104954062	0.81920896	Prevents CBL-SH3KBP1 complex facilitated down-regulation of EGFR signaling. Adapter protein involved in endocytosis and lysosomal degradation signaling pathways.
A0A087WQT6 Caspase−8	Casp8	41	0.586637451	0.032543796	Thiol protease, acts as a molecular switch for apoptosis, necroptosis and pyroptosis; *Salmonella* infection recruits caspase−8 to the inflammasome and regulates interleukin-1β production; *Salmonella* Typhimurium sseK3 induces caspase 8 activation and modulates macrophage apoptosis and glycolysis.
Q9D8U8 (Sorting nexin−5)	Snx5	90	0.537662356	0.032847912	Plays a role in various stages of intracellular trafficking; Essential for macropinocytosis and antigen processing; Activates endosomes and autophagy. SNX5 has been identified during proteomic analysis of RAW 264.7 macrophages infected with *Salmonella.*
Q91XV3 (Brain acid soluble protein 1)	Basp1	74	0.824853969	0.009652265	Interferes with the oncogenic capacity of MYC and its binding to calmodulin Proteomics analysis of Hela cells infection with *Salmonella* revealed that Basp1 is one of *Salmonella* ubiquitin ligase SlrP on host cells.
Q99PG2 (Opioid growth factor receptor)	Ogfr	46	0.636973632	0.024963872	Alternatively known as Met-enkephalin, has a role in growth regulation
P17439 (Lysosomal acid glucosylceramidase)	Gba1	24	0.581964936	0.020146627	Increase cholesterol glucosylation activity. Mutation of Gba1 is associated with Parkinson disease; catalyzes the hydrolysis of glucosylceramides within the lysosomal compartment
P97371 (Proteasome activator complex subunit 1)	Psme1	78	0.492696198	0.036703895	PA28 modulates antigen processing of viruses; Role in immunoproteasome assembly and essential for efficient antigen processing; *Salmonella* Typhimurium increased the expression of PA28 proteasome genes

**Table 5.** Functions of 18 downregulated proteins unique to E1-SifA conditions.

Uniprot ID/protein	Gene	Count	Fold change	*pv*alue	Function
E9QPK4 (Serine/threonine-protein kinase ATR)	Atr	17	−0.603450378	0.016430161	Promotes DNA repair after genomic stress *Salmonella enterica Serovar Typhi* secretes eukaryote-like Serine/Threonine Kinase for survival and pathogenesis
P63024 (Vesicle-associated membrane protein 3)	Vamp3	14	−0.827780184	0.006582661	Plays a role in vesicular transport from the late endosomes to the trans-Golgi network Involved in SCV inflation
Q9D1E8 (1-acyl-sn-glycerol−3-phosphate acyltransferase épsilon)	Agpat5	10	−0.931638085	0.006668647	Involved in lipid metabolism and cancer Converts 1-acyl-sn-glycerol−3-phosphate (lysophosphatidic acid or LPA) into 1,2-diacyl-sn-glycerol−3-phosphate
E0CZ72 (Kinesin-like protein)	Kif2a	40	−0.614010199	0.026019499	KIF2 is an anterograde motor, involved in fast axonal transport.
P97821 (Dipeptidyl peptidase 1)	Ctsc	10	−0.853043678	0.005275485	Thiol protease. Involved in the generation of cytotoxic lymphocyte effector functions. CTSC expression ↑ in S. Typhimurium-infected RAW264.7 cells and infected mice. It comes after proteomic analysis
Q99N84 (28S ribosomal protein S18b, mitochondrial)	Mrps18b	26	−0.881610909	0.019788747	Involved in cell stemness, differentiation, and carcinogenesis
A2AH22−2 (Isoform 2 of Activating molecule in BECN1-regulated autophagy protein 1)	Ambra1	41	−0.609281529	0.017959823	Ambra1 regulates mitophagy, autophagy, and development of nervous system.
B1AT66 (Monocarboxylate transporter 7)	Slc16a6	6	−1.076563712	0.003904615	Involved in transport of carboxylates, branched chain oxo acids across plasma membrane
Q8CHK3 (Lysophospholipid acyltransferase 7)	Mboat7	10	−1.004203972	0.012673109	Regulate phospholipids, arachidonic acid, triglyceride metabolism
A0A2I3BRL8 (Predicted gene 7324)	Gm7324	115	−0.661518175	0.014423047	Predicted gene
Q4VGL6 (Roquin−1)	Rc3h1	25	−0.755260065	0.021198993	Controls inflammation, role in maintenance of immune homeostasis. Post-transcriptional repressor of mRNAs, Cleaves translationally inactive mRNAs Regulates macrophage immune response and involved in hepatic ischemia reperfusion injury
A0A0N4SVL0 (Eukaryotic translation initiation factor 4 gamma 3)	Eif4g3	126	−0.637128776	0.040833242	EIF4G3 plays a role in mice spermatogenesis. EIF4G is broken-down by caspase 3 to inhibit cellular translation in apoptotic cells
Q8BJ48 (*N*-acetylglucosamine−1-phosphodiester alpha-*N*-acetylglucosaminidase)	Nagpa	28	−0.560997551	0.021936271	Involved in formation of the mannose 6-phosphate targeting signal on lysosomal enzymes
Q8BKF1 (DNA-directed RNA polymerase, mitochondrial)	Polrmt	17	−0.837935932	0.022028635	Involved in transcription of mitochondrial DNA into RNA.
Q9CRA8 (Exosome complex component RRP46)	Exosc5	16	−0.675175473	0.017455477	Non-catalytic component of the RNA exosome complex, role in RNA processing and degradation *Salmonella* infection reduced the levels of the exosome/NEXT components, RRP6 and MTR4, resulting in transcript (enhancer RNAs & long noncoding RNAs) stabilization
Q9CQ48 (NudC domain-containing protein 2)	Nudcd2	25	−0.61520271	0.022300646	Involved in LIS1/dynein pathway regulation
Q61103 (Zinc finger protein ubi-d4)	Dpf2	30	−0.846257937	0.028276294	Plays a role in the development and maturation of lymphoid cell
Q9Z0D9 (CX3C chemokine receptor 1)	Cx3cr1	3	−1.514025684	0.002246949	Receptor for the C-X3-C chemokine fractalkine (CX3CL1) present on many early leukocyte cells, role in immune response, inflammation, cell adhesion and chemotaxis *Salmonella* infection mediates migration of dendritic cells into the intestinal lumen via CX3CR1. CX3CR1 + Cell-Mediated *Salmonella* Exclusion Protects the Intestinal Mucosa during the Initial Stage of Infection. CX3CR1 + macrophage subsets recruit and activate CD4 + T and B cells to the sites of *Salmonella* invasion

Additionally, E1 cells infected with *SifA* presented 3 upregulated and 17 downregulated proteins that were common to pairs 9 and 10 (Figure 7B), suggesting the contribution of ELMO1 and SifA to the functions of these proteins. The upregulated proteins were associated with GO terms, including protein localization, lipid metabolism, and neutrophil activation while the downregulated proteins included biological processes related to cell differentiation, lipid metabolism, and organelle organization, among others. Interestingly, 11 upregulated and 2 downregulated proteins were common in Pair 7 and Pair 8 and were associated with biological processes related to mitochondrial fission and apoptosis, protein transport and secretion, and histone methylation.

## Discussion

Recent breakthroughs in omics technology identified several host signaling pathways following infection.^57^^,^^58^ Here, we performed liquid chromatography multinotch MS3-tandem mass tag (TMT) multiplexed proteomics to determine the global quantification of proteins regulated by ELMO1 in macrophages after *Salmonella* infection. We identified 7000 proteins and unique proteins exclusive to ELMO1-depleted cells after *Salmonella* (*SL*) infection or controlled by ELMO1 and the *Salmonella* effector SifA. ELMO1 regulates proteins with functions related to mitochondria, metabolism, vesicle transport, and the immune system. Our findings revealed that infection of macrophages with *Salmonella* profoundly altered cytosolic and mitochondrial metabolism, shifting it from oxidative phosphorylation to glycolysis, which aligns with previous studies that highlighted *Salmonella* stimulation of aerobic glycolysis in macrophages. This metabolic shift, accompanied by a reduced tricarboxylic acid (TCA) cycle and oxidative phosphorylation, facilitates *Salmonella* replication in host cells and mice.^42^^,^^43^ Importantly, the impact of *Salmonella*-induced changes in host metabolism, specifically glycolysis and oxidative phosphorylation, is accentuated by the depletion of ELMO1, indicating that ELMO1 is required for mitigating the effect of *Salmonella* on immunometabolism.

Mitochondria are important organelles that play a role in inducing apoptosis and maintaining immune homeostasis. Mitochondria regulate ATP production to meet cellular demands, calcium signaling, and antimicrobial reactive oxygen species (ROS) production—factors critical for eliciting an immune response to pathogenic infections.^59^ Therefore, pathogenic bacteria, including *Salmonella*, manipulate mitochondrial pathways to facilitate their growth. Our findings revealed a significant decrease in mitochondrial respiration and ATP production during *Salmonella* infection. We also demonstrated that the absence of ELMO1 after *Salmonella* infection reduced ATP production and mitochondrial respiration. Further investigations are needed to determine whether the changes after *Salmonella* infection are all ELMO1-dependent or ELMO1-independent.

Among the identified proteins, a significant number of host proteins linked with mitochondrial function as listed inTables 2–4. In addition to the proteins that we validated, the mitochondrial ribosomal proteins MRPL12, MRPL43, and MRPL49 were downregulated in E1 *SL* compared to C1 *SL*. Mitochondrial ribosomes (mitoribosomes) perform protein synthesis inside mitochondria, the organelles responsible for energy conversion and adenosine triphosphate production in eukaryotic cells. This finding provides an additional indication that ELMO1 plays a crucial role in controlling the overall functions of mitochondria. Dnm1l or DRP1 was the most upregulated (4-fold) protein in the ELMO1-depleted macrophages after *Salmonella* infection, as shown by the unique proteins inTable 2. We further confirmed that DRP1 is regulated translationally and increased in the ELMO1-depleted macrophage cell line as well as in the ELMO1-KO mouse line when we used the colonic tissue of mice infected with *SL*.

DRP1 is involved in mitochondrial fission.^49^^,^^50^ The coordinated balance between mitochondrial fusion and fission, known as mitochondrial dynamics, regulates mitochondrial morphology and function. Mitochondrial fission has primarily been linked to inflammatory responses and metabolic changes during specific energy demands, positioning it as a defensive strategy employed by the immune system. On the other hand, mitochondrial fusion plays a protective role by preventing cell death, particularly under nutrient starvation.^60^ Disruption of these mitochondrial mechanisms has been implicated in a range of conditions, including cancer, cardiovascular diseases and neurodegenerative disorders.^61-64^ Recent studies have highlighted the involvement of mitochondrial functions in gastrointestinal diseases, particularly inflammatory bowel diseases.^65^ Furthermore, mitochondrial dynamics and functions influence host‒pathogen interactions and host defenses by either promoting or limiting microbial survival and replication within host cells, depending on the pathogen and the cellular environment, as demonstrated in infection caused by *Listeria,*^66^ *Legionella*,^67^ *Helicobacter pylori* VacA^68^ and *Shigella* .^69^ The mitochondrial pathway can activate pattern-recognition receptors to initiate immune responses to clear pathogens. For example, TLR signaling triggers major changes in metabolic pathways in murine macrophages that connect TLR with mitochondrial function.^70^

The upregulation of proteins involved in mitochondrial fission and biogenesis in ELMO1-depleted macrophages after *Salmonella* infection suggests a putative role for ELMO1 in controlling mitophagy and other apoptotic pathways critical for mitochondrial health. Interestingly, the proteins differentially controlled by ELMO1-SifA are also associated with mitochondrial pathways and apoptosis (Nudt9 and Mrps18b), vesicle-mediated transport (SNX5), lipid metabolism, and regulation of the immune system. The link of these cellular signals with the *Salmonella* effector SifA will open new directions in host‒microbe interactions. Our findings indicate that *Salmonella* and other bacteria reprogram host metabolism and regulatory mechanisms inside immune cells to take advantage of the metabolites present inside the host and that these mechanisms are modulated by the host microbial sensor ELMO1.

## Conclusion

In the current work, we identified BOLA1 as one of the major downregulated mitochondrial proteins and DRP1 as one of the major upregulated mitochondrial proteins in the proteomic profiles of ELMO1-downregulated macrophages and in ELMO1 KO mice following *Salmonella* infection. Furthermore, our previous work on the ELMO1–SifA interaction that promotes bacterial colonization, dissemination, and the inflammatory immune response and the current work on the role of ELMO1–SifA in mitochondrial function and metabolism indicate a direct link between ELMO1 and controlling bacterial effectors to modulate host immune responses. Our work first indicated the importance of the major mitochondrial pathways connected to microbial sensor in infection and inflammatory responses which could be utilized in targeted therapies.

## Supplementary Material

Supplementary material Supplementary Figure Legends

## Data sharing plan

All proteomics data have been submitted, and all other raw data are accessible from the Cloud.

## Abbreviations

AGC: Automatic gain control
ANOVA: Analysis of variance
ATP: Adenosine triphosphate
BAI1: Brain Angiogenesis Inhibitor1
BOLA1: BolA Family Member 1
CID: collision-induced dissociation
DAVID: Database for Annotation, Visualization and Integrated Discovery
DEP: differentially expressed protein
DMEM: Dulbecco's Modified Eagle Medium
DOI: Digital Object Identifier
DRP1: Dynamin-related protein 1
ECAR: extracellular acidification rate
ELMO1: Engulfment and cell motility protein 1
ER: endoplasmic reticulum
FCCP: Carbonyl cyanide-p-trifluoromethoxyphenylhydrazone
GEF: guanine nucleotide exchange factor
GO: Gene Ontology
HCD: High Energy Collisional Dissociation
HEPES: (4-(2-hydroxyethyl)-1-piperazineethanesulfonic acid)
HRP: Horseradish Peroxidase
IAA: Iodoacetamide
IACUC: Institutional Animal Care and Use Committee
MS: Mass spectrometry
Myl6: myosin light chain peptide 6
NFkB: Nuclear factor kappa-light-chain-enhancer of activated B cells
OCR: oxygen consumption rate
OXPHOS: oxidative phosphorylation
PAMPs: pathogen-associated molecular patterns
PBS: Phosphate-buffered saline
PPI: protein-protein interaction
PRRs: Pattern Recognition Receptors
PVDF: polyvinylidene difluoride
RIG-1: Retinoic acid-inducible gene I
RIPA: Radioimmunoprecipitation assay buffer
ROS: reactive oxygen species
SCVs: Salmonella-containing vacuoles
SD: standard deviation
SDS/PAGE: Sodium dodecyl-sulfate polyacrylamide gel electrophoresis
SEM: standard error of the mean
SL: Salmonella
SNX5: Sorting Nexin 5
SPS: synchronous precursor selection
TCA: tricarboxylic acid cycle
TEAB: Triethylammonium bicarbonate
TFA: Trifluoroacetic acid
TGEb: transforming growth factor beta receptor signalling pathway
TNF-α: Tumor necrosis factor
TMT: Tandem Mass Tags
WT: Wild-type
