# Supplementary material for: Proteome profiling indicates a link between mitochondrial pathways and the host-microbial sensor ELMO1 following Salmonella infection
Source: Gut Microbes. 2025 Nov 17;17(1):2580708. doi: 10.1080/19490976.2025.2580708 (PMC12629339; doi:10.1080/19490976.2025.2580708)
Supplement: Supplementary material — Supplementary Figure Legends [file KGMI_A_2580708_SM3522.docx]

**Supplementary Figure Legends**

**
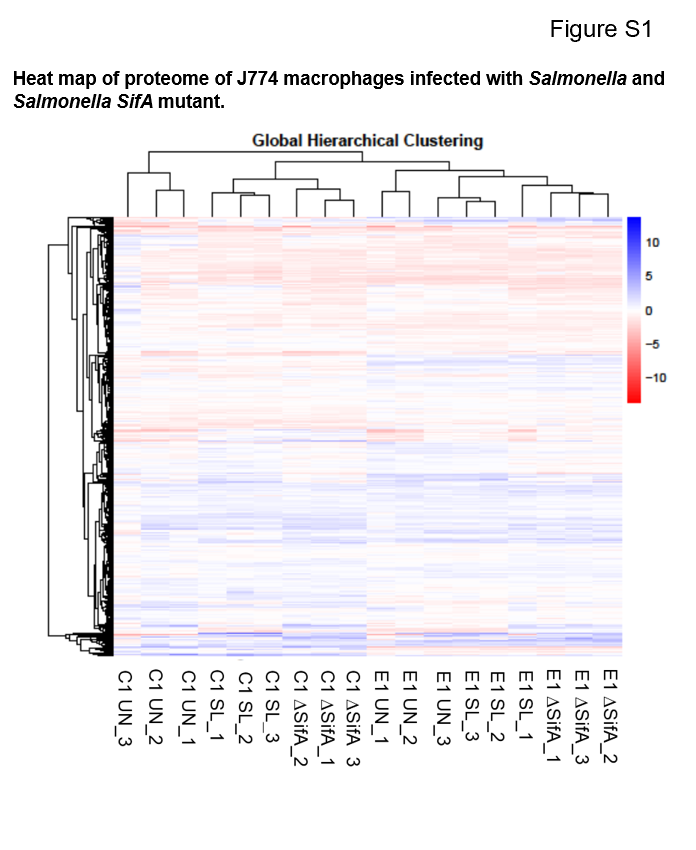
Figure S1**

A heatmap showing the total number of differentially expressed hits identified by mass spectrometry (blue color is used for upregulated targets and red color for downregulated targets). Different sample groups (in columns) were arranged using a clustering algorithm. The box at the bottom shows the total number of peptide spectral matches (200,148) and protein IDs (7777) identified.

**
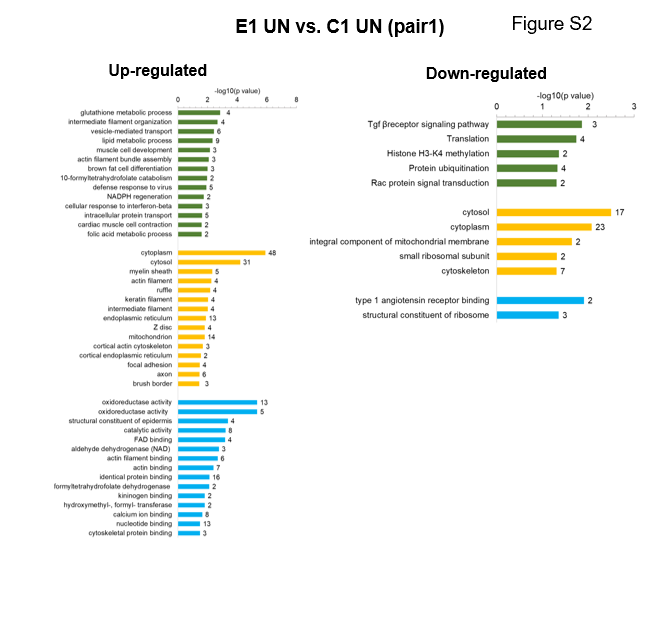
**

**Figure S2**

Representation of GO enrichment and DAVID Functional Annotations for analysis of 74 proteins upregulated proteins and 40 downregulated proteins in E1 UN vs C1 UN. Green Bars represent biological functions, yellow Bars represent cellular components, and blue bars represent molecular functions. The left panel represents upregulated proteins and the right panel represents downregulated ones.

**
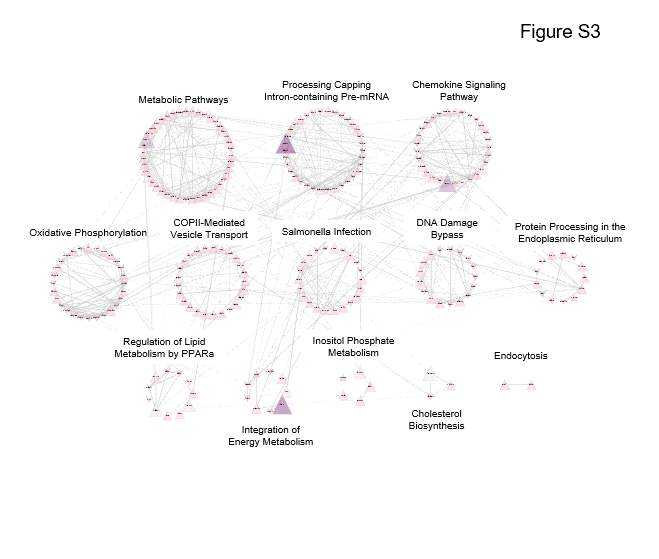
**

**Figure S3**

Protein-protein interaction (PPI) has been shown using STRING software to indicate pathways involved with ELMO1 following *Salmonella* infection.

**
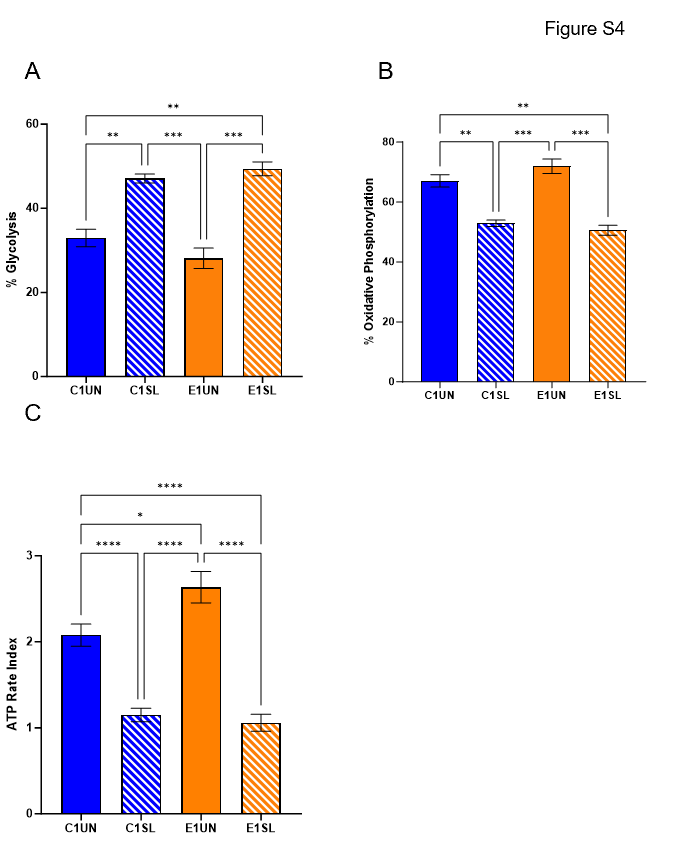
**

**Figure S4**

Quantification of the percentage of Glycolysis (A), percentage of Oxidative Phosphorylation (B), and ATP Rate Index (C) in C1 UN, E1 UN, and C1 and E1 following 6 h of infection with WT *SL* as measured by the instrument Agilent Seahorse XF HS Mini Analyzer. Ordinary one-way ANOVA was used for statistical analysis


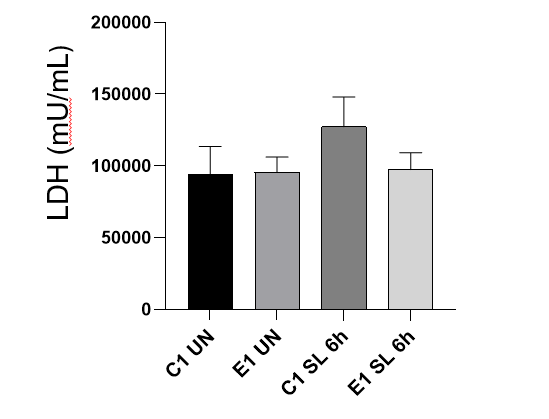
Figure S5

**Figure S5:** The amount of LDH is measured with the uninfected or infected C1-E1 cells following infection with SL from the previous experiments that used for proteomics. Data represented as mean ± SD of three separate experiments.

Figure S6

**
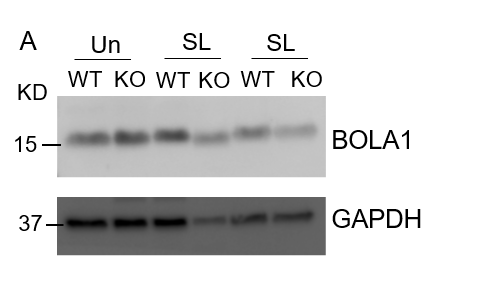
**

**
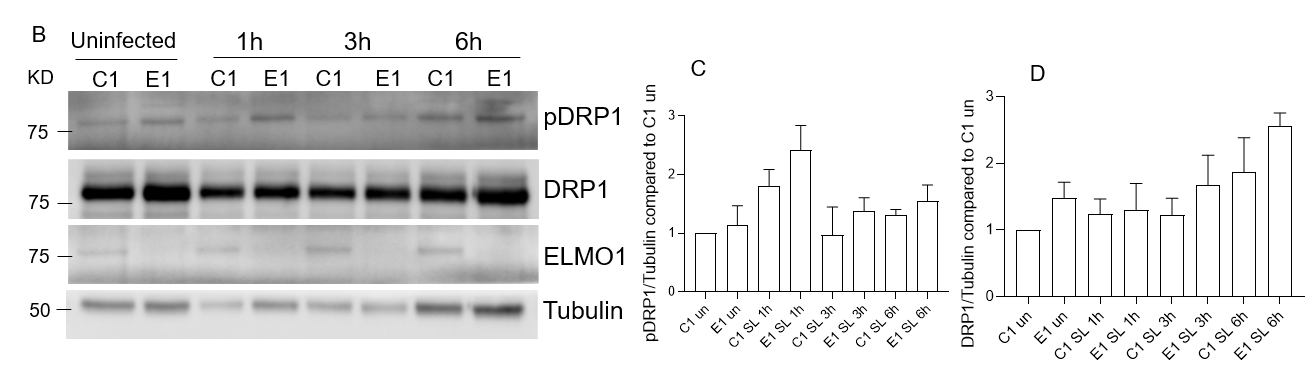
**

**Figure S6:** The impact of ELMO1 in the expression of BOLA1 and pDRP1 after *Salmonella* infection. (A) Western Blot of BOLA1 in the Ileum of WT and ELMO1 KO mice after 5 days of *SL* infection, as represented in Fig 4C. (B) The expression of pDRP1 in C1-E1 cells following infection with *SL* at different time point. (C-D) The densitometry was performed using two independent experiments and the data is normalized with Tubulin and compared with the uninfected C1 sample.

Figure S7

**
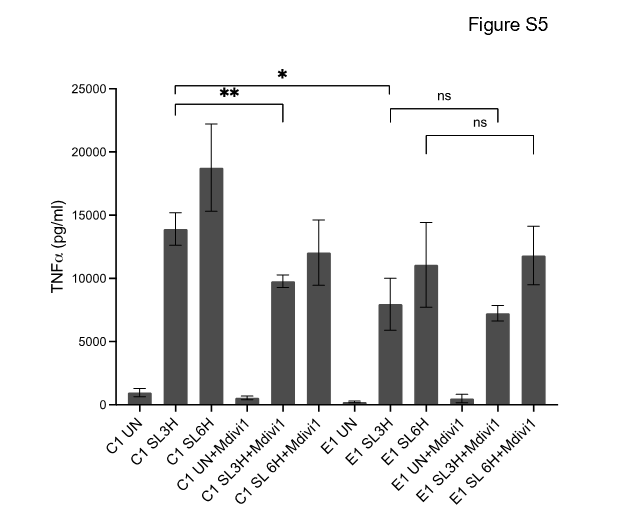
**

**Figure S7**

TNF-α cytokine levels in the supernatant from C1 and E1 cells after 3h and 6h infection with *SL* in the presence of DRP1 inhibitor mDivi-1, measured by ELISA. Data represent as the mean ± SEM of two separate experiments each including four biological replicates. * Indicates p ≤ 0.05, ** indicates p ≤ 0.01, as assayed by two-tailed Student's *t*-test.
